# HIF-PH inhibitors induce pseudohypoxia in T cells and suppress the growth of microsatellite stable colorectal cancer by enhancing antitumor immune responses

**DOI:** 10.1007/s00262-025-04067-3

**Published:** 2025-05-09

**Authors:** Yuehua Chen, Toshiaki Ohara, Yusuke Hamada, Yuze Wang, Miao Tian, Kazuhiro Noma, Hiroshi Tazawa, Masayoshi Fujisawa, Teizo Yoshimura, Akihiro Matsukawa

**Affiliations:** 1 Department of Pathology and Experimental Medicine, Graduate School of Medicine, Dentistry and Pharmaceutical Sciences, Okayama University, 2-5-1 Shikata-cho, Kita-ku, Okayama, 700-8558 Japan; 2 Department of Gastroenterological Surgery, Dentistry and Pharmaceutical Sciences, Okayama University Graduate School of Medicine, Okayama, Japan; 3 Center for Innovative Clinical Medicine, Okayama University Hospital, Okayama, Japan

**Keywords:** Colorectal cancer, Microsatellite stable, Hypoxia-inducible factor, Immune checkpoint inhibitors

## Abstract

**Background:**

Recent studies have revealed that CD8^+^ T cells can be activated via genetic upregulation of HIF-1α, thereby augmenting antitumor effector functions. HIF-1α upregulation can be attained by inhibiting HIF-prolyl hydroxylase (HIF-PH) under normoxic conditions, termed pseudohypoxia. This study investigated whether pseudohypoxia induced by HIF-PH inhibitors suppresses Microsatellite stable (MSS) colorectal cancer (CRC) by affecting tumor immune response.

**Methods:**

The HIF-PH inhibitors Roxadustat and Vadadustat were utilized in this study. In vitro, we assessed the effects of HIF-PH inhibitors on human and murine colon cancer cell lines (SW480, HT29, Colon26) and murine T cells. In vivo experiments were performed with mice bearing Colon26 tumors to evaluate the effect of these inhibitors on tumor immune responses. Tumor and spleen samples were analyzed using immunohistochemistry, RT-qPCR, and flow cytometry to elucidate potential mechanisms.

**Results:**

HIF-PH inhibitors demonstrated antitumor effects in vivo but not in vitro. These inhibitors enhanced the tumor immune response by increasing the infiltration of CD8^+^ and CD4^+^ tumor-infiltrating lymphocytes (TILs). HIF-PH inhibitors induced IL-2 production in splenic and intratumoral CD4^+^ T cells, promoting T cell proliferation, differentiation, and immune responses. Roxadustat synergistically enhanced the efficacy of anti-PD-1 antibody for MSS cancer by increasing the recruitment of TILs and augmenting effector-like CD8^+^ T cells.

**Conclusion:**

Pseudohypoxia induced by HIF-PH inhibitors activates antitumor immune responses, at least in part, through the induction of IL-2 secretion from CD4^+^ T cells in the spleen and tumor microenvironment, thereby enhancing immune efficacy against MSS CRC.

**Supplementary Information:**

The online version contains supplementary material available at 10.1007/s00262-025-04067-3.

## Introduction

Colorectal cancer (CRC) stands as a prevalent ailment and ranks as the second leading cause of cancer-related mortality worldwide [1]. Immunotherapy emerges as a significant advancement in CRC treatment [2]. Mismatch repair (MMR) deficient tumors accrue numerous somatic mutations, including indels in microsatellite repeats, resulting in a distinct molecular phenotype termed microsatellite instability (MSI). While patients with MSI-high CRC exhibit favorable responses to immunotherapy, including immune checkpoint inhibitors (ICIs), those with microsatellite stable (MSS) CRC, constituting the majority, are unresponsive to ICIs [3]. Therefore, there is a pressing need for novel therapeutic approaches to enhance immunotherapy for MSS CRC patients.

Hypoxia-inducible factor (HIF) serves as a vital transcription factor, playing a crucial role in the body's adaptation to low oxygen levels, known as hypoxia. HIF upregulation promotes angiogenesis to counteract ischemic conditions [4]. In the oncologic setting, hypoxia is a defining feature of the tumor microenvironment (TME) and a critical driver of cancer hallmarks. Hypoxia modulates both immune and non-immune components within the TME, facilitating immune evasion and therapy resistance [5]. Severe hypoxia is a principal contributor to resistance against PD-1/PD-L1 inhibitor therapies. HIF-1 under hypoxic condition induces a state of terminal exhaustion in CD8^+^ T cells, characterized by the expression of CD39, a marker of this exhausted phenotype. Furthermore, hypoxia stimulates the secretion of vascular endothelial growth factor (VEGF) from terminally exhausted CD8^+^ T cells, promoting their differentiation into a fully exhausted state [5]. CD8^+^ T cells exposed to 1% oxygen (hypoxia) have been shown to exhibit enhanced maturation, survival, and cytotoxicity against cancer cells [6]. However, the expression dynamics of HIF-1α under normoxia remain elusive. Recent studies have demonstrated that upregulating HIF-1α in CD8^+^ T cells through VHL knockdown enhances cytotoxic function and suppresses tumor growth [7]. HIF expression is tightly regulated through hydroxylation by HIF-prolyl hydroxylase (HIF-PH) in response to hypoxia, with iron serving as a catalyst [8]. Inhibition of HIF-PH activity by iron chelators are expected to boost tumor immune response by upregulating HIF in CD8^+^ T cells, akin to VHL knockdown. However, HIF-PH inhibitors carry the risk of increasing HIF expression in cancer cells, thereby promoting tumor growth with enhancing angiogenesis [9]. It remains uncertain whether HIF-PH inhibitors with iron-chelating activity suppress tumor growth by activating tumor immune responses.

HIF-PH inhibitors such as Roxadustat and Vadadustat, used in the treatment of anemia related to chronic kidney disease (CKD) by enhancing HIF expression targeting the EPO gene under normoxia [10, 11]. Under conditions of HIF-PH inhibition, HIF expression increases even in the presence of normal oxygen levels, a state referred to as pseudohypoxia. This study investigates whether pseudohypoxia induced by HIF-PH inhibitors suppresses tumor growth by activating tumor immune responses and elucidates the underlying mechanisms.

## Materials and methods

All experiments involving animals were conducted according to the ethical policies and procedures approved by The Animal Care and Use Committee of Okayama University (Approved No. OKU-2021900, OKU- 2022723, OKU- 2022879, OKU- 2023535). Details about materials and methods are provided in Supplemental Information.

## Results

### *HIF-PH inhibitors exhibited anti-tumor effects *in vivo*, but not *in vitro

**Fig. 1:**
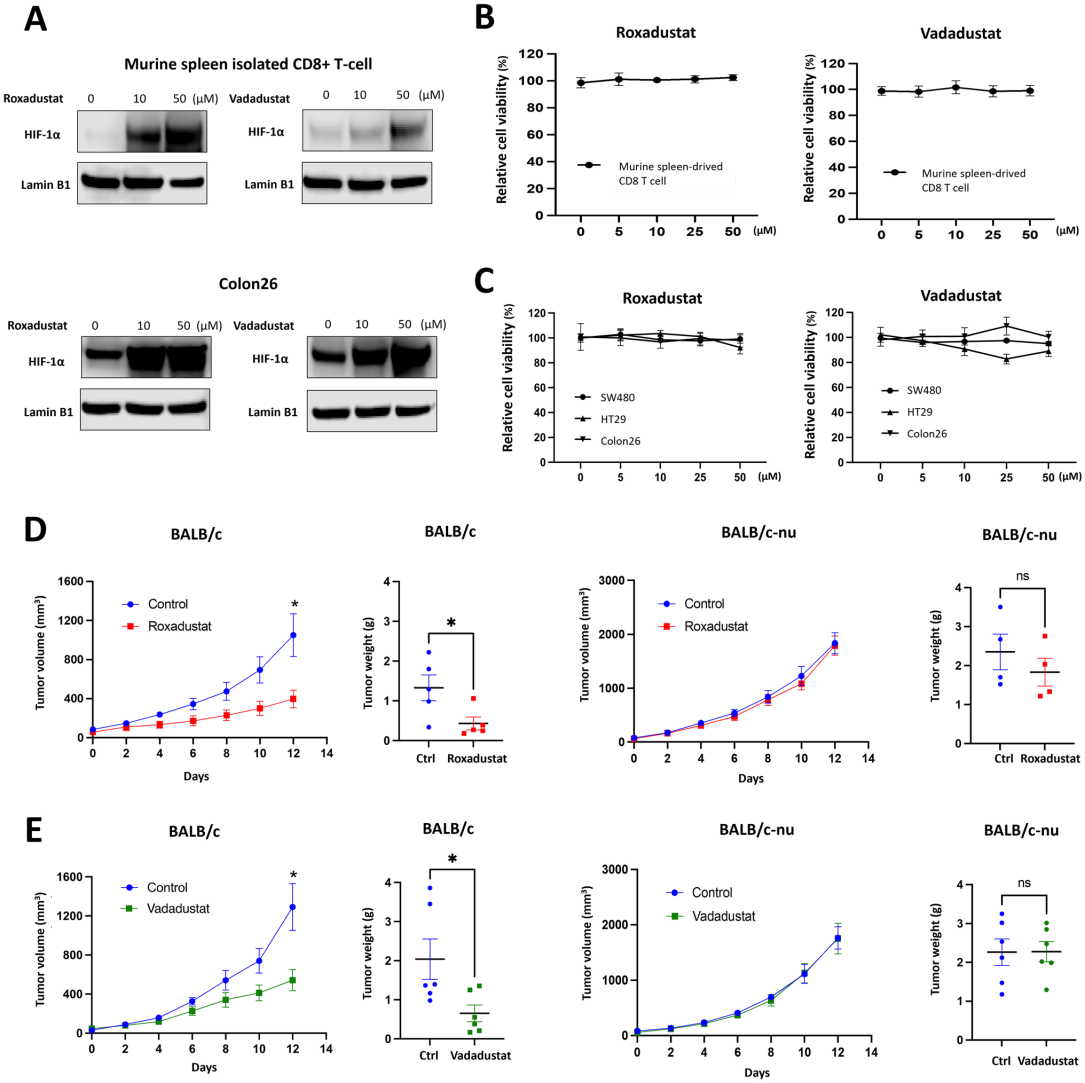
HIF-PH inhibitors activate immune system to suppress the tumor. **A** Murine spleen-derived CD8^+^ T cells and Colon26 were treated with different HIF-PH inhibitors for 48 h. HIF-1α was measured using western blotting. **B**, **C** Murine spleen-derived CD8^+^ T cells and MSS colon cancer cell lines (SW480, HT29, Colon26) were treated with different concentrations of HIF-PH inhibitors for 48 h. Cell viability was assessed by XTT assay. **D**, **E** 7 days post Colon26 injection, BALB/c and BALB/c-nude mice were randomized into two groups: (i) Control; (ii) Roxadustat/Vadadustat; PBS and Roxadustat/Vadadustat were administered via oral route (Roxadustat: 50 mg/kg. every other day. Vadadustat: 150 mg/kg. every day.). Tumor volumes and weights of BALB/c and BALB/c-nude mice were measured at the indicated day under HIF-PH inhibitors treatment (Roxadustat’s n = 5, Vadadustat’s n = 6). The results are presented as the means ± S.E.M. of a representative experiment performed in triplicate. Student’s t test. *P < 0.05, **P < 0.01, ***P < 0.001

To verify the HIF-1α upregulation by HIF-PH inhibitors, the expression of HIF-1α in CD8^+^ T cells and colon cancer cells was evaluated following treatment with Roxadustat and Vadadustat, both of which possess iron-chelating properties. Roxadustat exhibited stronger iron chelating ability (Fig. S1A-B) and induced greater HIF-1α expression compared to Vadadustat (Fig. 1A and S1C). Importantly, neither Roxadustat nor Vadadustat affected the proliferation of CD8 ^+^ T cells or MSS colon cancer model cells, including human SW480 and HT29 cells and the murine Colon26 cells (Fig. 1 B-C and S1D) [12, 13]. To examine the in vivo effects of HIF-PH inhibitors, we employed the subcutaneous Colon26 tumor transplantation model. Roxadustat notably suppressed the tumor growth in immunocompetent BALB/c mice, but this effect was abrogated in BALB/c nude mice (Fig. 1D). The BALB/c nude mice, characterized by a pivotal mutation in the Foxn1 gene, exhibit defective thymus development and a consequent lack of mature T cells [14]. Vadadustat yielded similar outcome (Fig. 1E). Since HIF-PH inhibitors do not directly affect cancer cell proliferation, these findings imply that observed tumor growth suppression is mediated by immune responses.

### *HIF-PH inhibitors enhanced tumor immune response by increasing TIL infiltration*

**Fig. 2:**
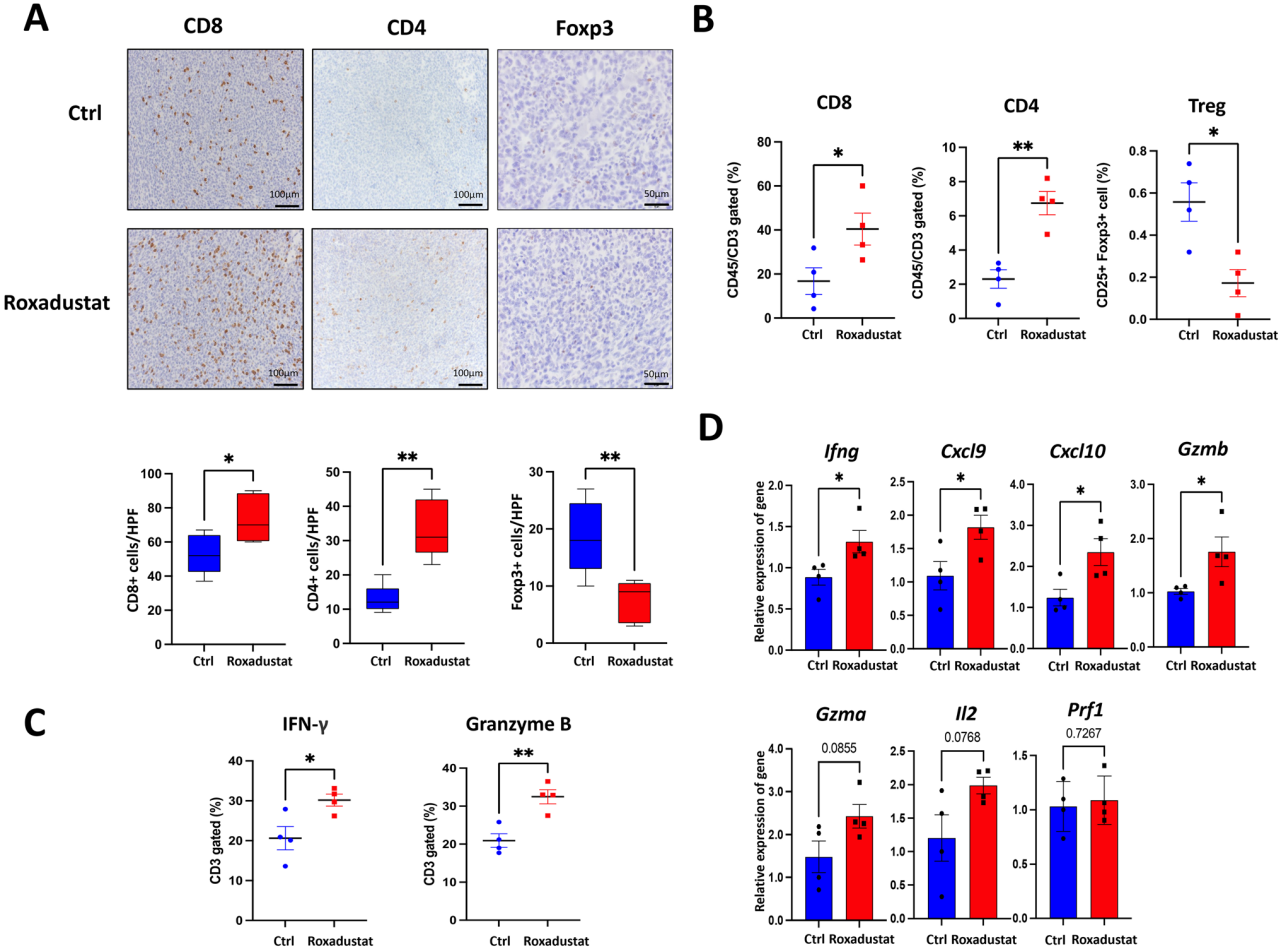
HIF-PH inhibitors enhance T cell efficacy, promote CD4^+^ and CD8^+^ T cell infiltration, and reduce regulatory T cell populations. **A** Representative images and positive cell number of CD8^+^, CD4^+^ and Foxp3^+^ cells stained from Colon26 tumor tissues of control and Roxadustat treatment group mice. The number of positive cells was manually counted from five selected high-power fields (HPFs) per tumor. (n = 5; scale bar,100 and 50 μm; quantitative data, blow). **B**, **C** Frequencies of the indicated cells (n = 4) for TME collected from tumor-bearing mice. **D** The mRNA levels of indicated markers were measured by RT-qPCR. Total mRNA was extracted from Colon26 tumor tissues (n = 4). The results are presented as the means ± S.E.M. of a representative experiment performed in triplicate. Student’s t test. *P < 0.05, **P < 0.01, ***P < 0.001

To analyze immune cell populations in the TME, collected tumors were analyzed by immunohistochemistry (IHC) and flow cytometry. Roxadustat treatment significantly increased both the number and percentage of CD8^+^ and CD4^+^ tumor-infiltrated lymphocytes (TILs) (Fig. 2A-B). The treatment also decreased the number and percentage of Foxp3^+^ regulatory T cells (Tregs). A previous study suggested HIF-1α could attenuate Treg cell differentiation by binding to and accelerating the degradation of Foxp3 protein, and our results are consistent with the report [15]. Similar outcomes were observed with Vadadustat (Fig. S2A). The number of Foxp3^+^ cells in the control group determined by IHC was slightly higher than that of CD4^+^cells, likely due to the heterogeneous distribution of Foxp3⁺ cells within the tumor tissue [16, 17]. Additionally, the number of T-bet-positive cells, a characteristic marker of CD4⁺ Th1 cells, was increased in the Roxadustat group, suggesting that Roxadustat also promotes the expansion of immune-enhancing CD4⁺ T cell subsets (Fig. S2B). Furthermore, flow cytometry revealed increased percentages of IFN-γ^+^ and Granzyme B^+^ CD3^+^ T cells in the Roxadustat group (Fig. 2C). Reverse transcription quantitative PCR (RT-qPCR) analysis of tumor tissues unveiled a significant upregulation of several immune-related genes, including *Ifng, Gzmb, Cxcl9* and *Cxcl10* (Fig. 2D). Furthermore, peripheral blood analysis of mice showed an increased number of white blood cells (WBC), supporting the enhanced TILs infiltration (Table S3). These results suggest that HIF-PH inhibitors promote the recruitment and activation of TILs through increased production of chemokine and cytokine, thereby enhancing antitumor immune responses.

### ***HIF-PH inhibitors induced IL-2 production in splenic CD4***^+^***T cell, promoting T cell proliferation, differentiation, and immune response***

**Fig. 3:**
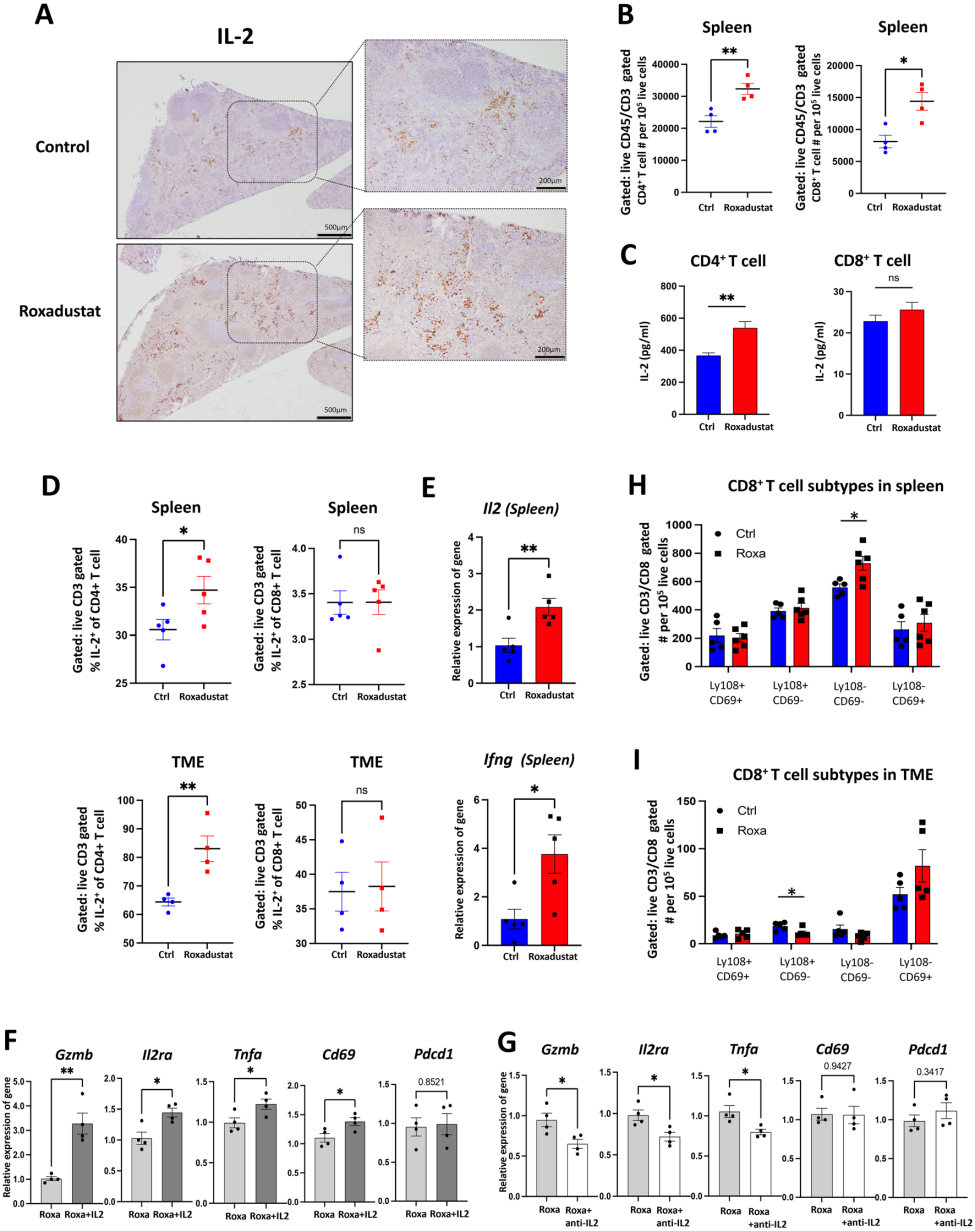
HIF-PH inhibitors elevate IL-2 levels in CD4^+^ T cell to boost T cell proliferation and CD8^+^ T cell activation. **A** Representative images of IL-2 protein in tumor-bearing mice spleen of control and Roxadustat group mice (scale bar, 500 μm; magnification scale bar, 200 μm). **B** Numbers of the indicated cells (n = 4) for spleen collected from tumor-bearing mice. **C** Concentrations of IL-2 from indicated murine spleen-derived cells stimulated with 5 μg/ml ConA and treated with 25 μM Roxadustat for 48 h. **D** IL-2 frequencies of the indicated cells (n = 4) for spleen and TME collected from tumor-bearing mice. **E** The mRNA levels of indicated markers were measured by RT-qPCR. Total mRNA was extracted from spleen of tumor-bearing mice (n = 5). **F** Isolated CD8⁺ T cells were stimulated with 5 μg/ml ConA and treated with 5 μM Roxadustat in the presence or absence of 50 ng/ml recombinant IL-2 for 12 h. mRNA expression levels were analyzed by RT-qPCR. **G** Isolated CD8⁺ T cells were cultured for 12 h in conditioned media (supernatants) collected from CD4⁺ T cells pre-treated with 5 μg/ml ConA and 5 μM Roxadustat, in the presence or absence of 2.5 μg/ml anti-IL-2 antibody. mRNA expression levels were analyzed by RT-qPCR. **H, I** Numbers of different CD8^+^ T cell subtypes (n = 5) for spleen and TME collected from tumor-bearing mice. The results are presented as the means ± S.E.M. of a representative experiment performed in triplicate. Student’s t test. *P < 0.05, **P < 0.01, ***P < 0.001

As described above, RT-qPCR analyses of tumor tissues and splenic CD4^+^ T cells revealed an upward trend in IL-2 expression, prompting further investigation into IL-2 expression within the lymphatic system (Fig. 2D and S3A). IL-2, a key T cell growth factor, promotes not only T cell proliferation but also the differentiation of CD8^+^ T cells into more potent cytotoxic subtypes [18]. Based on the RT-qPCR results, spleens were collected, and the expression of IL-2 was examined by IHC, RT-qPCR and flow cytometry. Roxadustat treatment enhanced IL-2 staining intensity and notably increased the number of CD4^+^ and CD8^+^ T cells in the spleens (Fig. 3A-B). To identify the cellular source of IL-2 in the spleen, CD4⁺ and CD8⁺ T cells isolated from murine spleens were analyzed for IL-2 secretion by ELISA in vitro. Both CD4^+^ and CD8^+^ T cells were examined due to reports that IL-2 can also be secreted by CD8^+^ T cells, although the primary source is CD4^+^ T cells [19]. ELISA results confirmed that Roxadustat significantly increased IL-2 secretion from isolated splenic CD4^+^ T cells (Fig. 3C). To validate these in vitro results, intracellular IL-2 expression in CD4⁺ and CD8⁺ T cells from the spleen and tumor was analyzed by flow cytometry. Roxadustat treatment increased the percentage of IL-2⁺ CD4⁺ T cells, but not CD8⁺ T cells, in both the spleen and TME (Fig. 3D). Consistently, RT-qPCR analysis of spleens revealed upregulated expression of *Il2* and *Ifng* following Roxadustat treatment (Fig. 3E). Similar results were observed with Vadadustat (Fig. S3B-C). These findings suggest that HIF-PH inhibitors promote IL-2 production specifically in CD4⁺ T cells.

We next explored the functional relevance of IL-2 in modulating CD8⁺ T cell activation through IL-2 supplementation and neutralization assays. RT-qPCR analyses revealed that IL-2 supplementation upregulated the expression of activation markers such as *Cd69, Tnfa,* and *Il2ra* (the IL-2 receptor alpha chain), along with an elevated level of the cytotoxic effector molecule granzyme B (*Gzmb*). In contrast, expression of *Pdcd1* (encoding PD-1), an exhaustion marker, remained unchanged (Fig. 3F). These findings suggest that IL-2 derived from activated CD4^+^ T cells has the capacity to promote CD8^+^ T cell activation. Conversely, IL-2 blockade attenuated the expression of these activation-related genes, while *Cd69* and *Pdcd1* levels remained relatively unaffected (Fig. 3G). These findings suggest that IL-2 secreted by CD4^+^ T cells under HIF-PH treatment is a key driver of CD8^+^ T cell activation, although other activation signals may also contribute.

Moreover, IL-2 secretion may support the enhancement of tumor immune responses. To assess CD8⁺ T cell phenotypes, flow cytometry was performed on cells from the spleen and tumor (Fig. 3H-I). CD8^+^ T cells negative for Ly108 and CD69, markers of effector-like transitory CD8^+^ T cells [20, 21], were significantly increased in spleen treated by Roxadustat (Fig. 3H). Additionally, the proportion of Ly108^+^ CD69^−^ CD8⁺ T cells, markers of stem-like progenitor CD8^+^ T cells, was significantly decreased (Fig. 3I). This suggests that Roxadustat facilitates the accumulation of effector-like CD8^+^ T cells in the spleen and promotes the differentiation of stem-like CD8^+^ T cells, thereby potentially priming subsequent antitumor immune responses. Furthermore, the percentage of Ly108^−^ and CD69^+^ T cells, which are characteristic of exhausted CD8⁺ T cells that still retain significant cytotoxic potential [20], was also high in the tumor. This phenotypic shift in both the spleen and TME indicates effective tumor targeting by cytotoxic CD8⁺ T cells, potentially supported by IL-2 secretion from CD4⁺ T cells in both compartments. Collectively, these results suggest that HIF-PH inhibitor induces IL-2 derived from CD4⁺ T cells to enhance antitumor immunity by promoting the functional activation and differentiation of cytotoxic CD8⁺ T cells.

### *Roxadustat enhances the efficacy of Anti-PD-1 antibody for MSS cancer with increasing TILs*

**Fig. 4:**
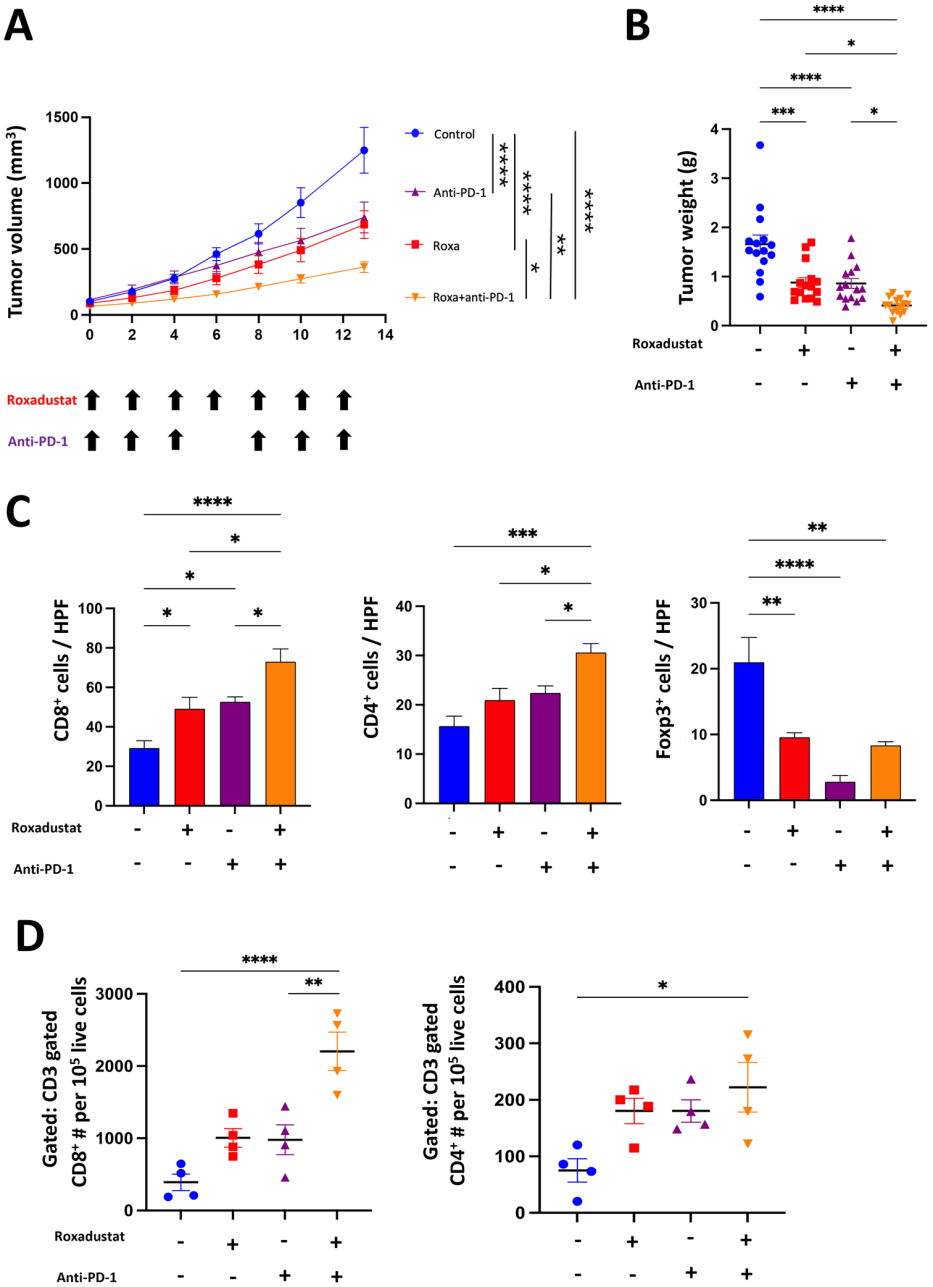
Roxadustat and anti-PD-1 combination therapy enhances MSS tumor suppression and increase TILs. **A**–**D** 7 days after colon 26 inoculation, BALB/c mice were randomized into four groups: (i) Control; (ii) Roxadustat; (iii) aPD1; (iv) Roxadustat + aPD-1. Roxadustat (50 mg/kg) was administered orally, and aPD-1(7 mg/kg) intraperitoneally. (A-B) Tumor growth and weight of Colon26 tumors in BALB/c mice treated with PBS, Roxadustat, aPD-1, or Roxadustat + aPD-1. (n = 15/ group pooled from three experiments). **C** IHC was used to analyze the staining of CD8^+^, CD4^+^ and Foxp3^+^ cells for TME collected from tumor-bearing mice (n = 5). The number of positive cells was counted manually. **D** Numbers of CD4^+^ and CD8^+^ T cells (n = 5) for TME collected from tumor-bearing mice were measured by flow cytometry. The results are presented as the means ± S.E.M. of a representative experiment performed in triplicate. ANOVA followed by Tukey’s multiple comparison test was applied. *P < 0.05, **P < 0.01, ***P < 0.001

In clinical scenarios, MSS tumors are often characterized by a dearth of mutant neoantigens, resulting in insufficient immune responses. These tumors have demonstrated a diminished response to immune check point inhibitors (ICIs), as evidenced by shorter progression-free survival (PFS) and overall survival (OS) [22, 23]. Considering the results of our in vivo studies, HIF-PH inhibitors were expected to enhance antitumor immune responses and improve the efficacy of ICIs. We therefore evaluated the combination therapy of Roxadustat and anti-PD-1 antibody in a subcutaneous Colon26 tumor model. The combination therapy significantly suppressed the tumor growth compared with either monotherapy (Fig. 4A-B). The number of CD8^+^ and CD4^+^ TILs was significantly increased in the combination group (Fig. 4C-D and S4A). The number of Foxp3-positive cells in the combination group was similar to that in the Roxadustat monotherapy group (Fig. 4C). These results indicate that Roxadustat enhances the efficacy of anti-PD-1 therapy in MSS tumors by increasing the infiltration of TILs.

### ***Synergistic increase of effector-like CD8***^+^***T cell population by Roxadustat combined with anti-PD-1 therapy***

**Fig. 5:**
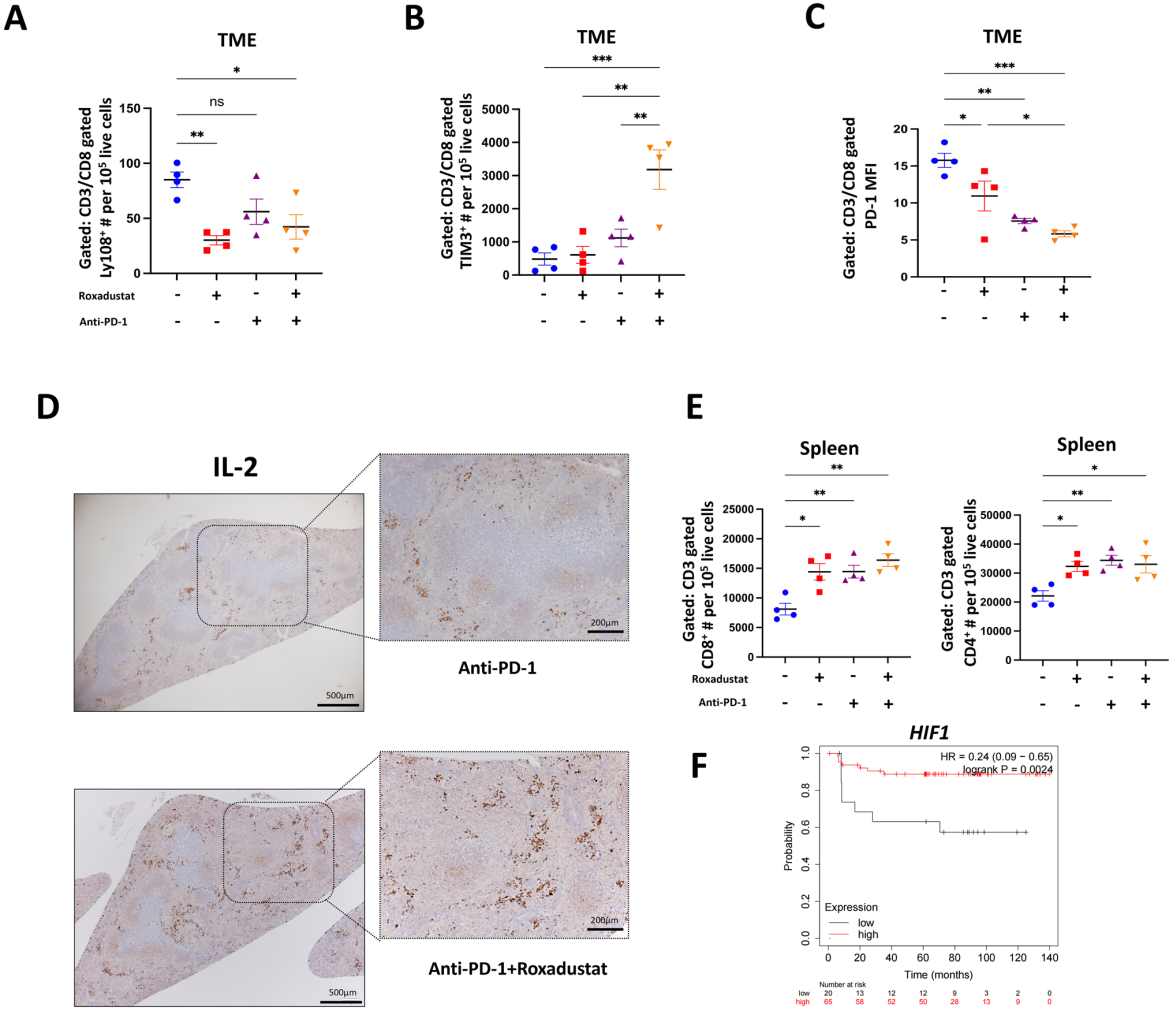
Synergistic increase of effector-like CD8^+^ T-cell population by Roxadustat combined with anti-PD-1 therapy. 14 days post treatment initiation, the indicated tissues from tumor-bearing BALB/c mice were examined by flow cytometry (n = 4/group). **A**, **B** Numbers of the indicated subtypes for CD8^+^ T cells collected from TME of tumor-bearing mice. **C** PD-1 MFI of CD8^+^ T cells collected from TME of tumor-bearing mice. **D** Representative images of IL-2 protein in tumor-bearing mice spleen of control and Roxadustat group mice (scale bar, 500 μm; magnification scale bar, 200 μm). **E** Numbers of the indicated T cells collected from spleen of tumor-bearing mice. **F** Kaplan–Meier shows progression-free survival of patients with MSS colon cancer classified by stable MSI and the level of *HIF-1* expression: high (red) and low (black), measured with Affymetrix arrays (IDs: 220946_s_at). Data were from GSE143985 in GEO database. The results are presented as the means ± S.E.M. of a representative experiment performed in triplicate. ANOVA followed by Tukey’s multiple comparison test was applied. *P < 0.05, **P < 0.01, ***P < 0.001

To elucidate the mechanism of enhanced antitumor effects obtained by the combination therapy, detailed T cell phenotype in the tumor and spleen was analyzed. The number of CD8^+^ T cells expressing Ly108, indicative of stem-like CD8^+^ T cells [21], was significantly reduced in the Roxadustat and combination group (Fig. 5A). Additionally, the number of CD8^+^ T cells expressing Tim3 as well as Ly108^−^ PD1^low^ and Tim3^+^ PD1^low^, a representative marker of effector-like CD8^+^ T cells [21, 24], was markedly increased in the combination group (Fig. 5B and S5A-B). Furthermore, the mean fluorescence intensity (MFI) of the exhaustion marker PD-1 on CD8⁺ T cells was reduced in all treatment groups, with the most pronounced decrease observed in the combination group, suggesting enhanced cytotoxic potential of CD8⁺ T cells following combination therapy (Fig. 5C). These findings suggested that the combination therapy induced both the proliferation and phenotypic transition of CD8^+^ T cells from a stem-like to an effector-like state within the TME. To verify the underlying mechanism, increased IL-2 staining intensity was confirmed by IHC in the combination therapy group compared to anti-PD-1 monotherapy group (Fig. 5D and S5C). In parallel, the numbers of CD8^+^ and CD4^+^ T cells were also increased in the spleen (Fig. 5E).

In a clinical context, database analysis revealed that high *HIF-1* expression in MSS cancer patients correlated with improved prognosis compared to low expression, further corroborating our findings (Fig. 5F). Together, these results suggest that the combination therapy of Roxadustat and anti-PD-1 antibody promotes the induction of effector-like CD8^+^ T cells within the tumor, a process supported by Roxadustat-induced increased IL-2 production. This outcome is attributed to both the induction of effector-like CD8^+^ T cells by anti-PD-1 antibody and the differentiation of CD8^+^ T cells facilitated by Roxadustat.

## Discussion

Our discussion highlights that pseudohypoxia, induced by HIF-PH inhibitors, leads to an increase in HIF-1α levels in both immune and tumor cells under normoxic conditions. Although concerns were raised that elevated HIF-1α levels might facilitate tumor growth via VEGF upregulation, the activation of the tumor immune response mitigated this risk, resulting in tumor growth inhibition. HIF-1α is recognized as a promoter of tumor progression, and substantial efforts have been directed towards developing HIF inhibitors as therapeutic agents due to their upstream regulation of VEGF. However, most of these attempts have been unsuccessful [25]. There is compelling evidence supporting our hypothesis in both immune and cancer cells. The expression of HIF-1α is regulated by Von Hippel-Lindau (VHL) and factor inhibiting HIF (FIH). Both VHL and FIH modulate cellular responses to oxygen levels, adapting to hypoxia: their knockdown increases HIF-1α expression. CD8^+^ T cells with high HIF-1α expression due to VHL^KO^ or FIH^KO^ were reported to enhance the effector response to persistent antigen [26, 27]. Additionally, FIH^KO^ in cancer cells, particularly lung cancer, was shown to suppress tumor growth in an orthotopic lung tumor model by increasing angiomotin (Amot), a key component of the Hippo tumor suppressor pathway [28]. Conversely, HIF-1α depletion was reported to disrupt T cell receptor activation and antitumor T cell function, providing opposing evidence [29, 30].

In the spleen, we observed a significant increase in IL-2 level. IL-2 is a well-established immune-activating cytokine, particularly in T cell-mediated immunity [31]. IL-2 promotes the proliferation and differentiation of T cells, crucial for an effective immune response [32, 33]. However, IL-2 supplemental therapies, such as IL-2 analogues, were reported to increase Treg populations [34, 35]. Interestingly, HIF-1α impedes Treg differentiation by binding to and hastening the degradation of the Foxp3 protein, which contribute to increased TILs [15]. Indeed, IL-2 induction by HIF-PH inhibitors appears superior in reducing Treg level compared to other IL-2 therapies. These findings support the notion that HIF-1α upregulation therapy, including HIF-PH inhibitors, exerts significant antitumor effects, corroborating our observations. While previous studies focused on HIF regulation through genetic manipulation in immune or cancer cells, our study demonstrate that HIF-PH inhibitors can enhance antitumor responses without such manipulation.

Our findings revealed that CD8^+^ T cell activation is mediated, at least in part, by CD4⁺ T cell-derived IL-2. Anti-PD-1 therapy was reported to provide a significant proliferation of stem-like CD8^+^T cells [36]. Additionally, our results showed a notable increase in effector-like CD8^+^ T cells in the spleen and decrease of stem-like CD8^+^ T cells in the tumor, which indicates HIF-PH inhibitors could promote CD8^+^ T cell differentiation. This shift indicates that activated CD8^+^ T cells effectively attacked cancer cells in the TME, which was supported by an increased number and activation of CD4^+^ and CD8^+^ T cells in the spleen. Collectively, pseudohypoxia induced by HIF-PH inhibitors enhances the tumor immune response via IL-2 induction by CD4^+^ T cells in the spleen and TME. Moreover, recent studies have provided additional insights into the functional characteristics of these subsets. Ly108⁻ TIM3⁺ terminal CD8⁺ T cells produce significantly higher levels of IFN-γ, TNF, and Granzyme B compared to Ly108⁺ TIM3⁻ stem-like progenitor CD8⁺ T cells [24]. Similarly, TIM3⁺Ly108⁻ terminally dysfunctional CD8⁺ T cells also exhibit high Granzyme B expression [37]. Based on the insights, a dual-marker analysis revealed a significant rise in effector-like transitory CD8^+^ T cells, characterized as PD-1^low^ and TIM3^+^ or PD-1^low^ and Ly108^−^ within the combination therapy group (Fig. S5A-B).

Iron chelation is pivotal in increasing HIF-1α expression for antitumor immune responses as shown here and also by inhibiting the progression of cancer cell malignancy [38]. Iron is essential for both normal and cancer cell proliferation. Iron chelators have been reported to suppress the proliferation and function of various cancer cells, particularly cancer stem cells (CSCs) [39, 40]. While iron chelation or depletion generally does not enhance cancer cell proliferation and function, its effect on tumor immune responses remains unclear. This study provides new insights indicating that pseudohypoxia induced by iron chelators can activate the tumor immune response, suggesting potential pharmaceutical targets. Our new evidence is expected to contribute to establishing pseudohypoxia induced by iron chelation as a novel approach to cancer immunotherapy.

Regarding drug selection, dosage, and clinical applicability, careful consideration is required. Although the dosages administered to mice were selected based on prior studies, the clinically relevant dosage in humans is substantially lower [41–45]. Despite efforts to reduce the dosage, the outcomes were insufficient (Fig. S6A). The approved dosages of Roxadustat and Vadadustat for humans are significantly lower compared to those used in mice [46, 47]. The safety of HIF-PH inhibitors for MSS CRC patients remains unclear, despite Roxadustat and Vadadustat being clinically available for renal anemia patients. Moreover, the duration for which HIF-1α expression is sustained within the tumor remains unclear. Western blot and IHC analyses indicated that HIF-1α expression was markedly elevated in the once-daily Vadadustat group but not significantly in the once-every-two-days Roxadustat group (Fig. S6B-C). This difference may be attributed to the intrinsic instability and rapid degradation of HIF-1α, suggesting the possibility that long-acting iron chelators may robustly induce HIF-1α in tumors, thereby exerting a tumor-suppressive effect. Furthermore, in our animal experiments conducted at the tested concentrations, no adverse mortality cases were observed. Thus, this study suggests a possibility that HIF-PH inhibitors have the potential, in principle, to activate antitumor immunity.

The TME comprises various cell types and substances, and there are significant considerations regarding hypoxia and pseudohypoxia. For instance, hypoxic extracellular vesicles (EVs) play a central role in cancer progression by driving M2 macrophage polarization, expanding myeloid-derived suppressor cells (MDSCs), inducing epithelial-to-mesenchymal transition (EMT), facilitating angiogenesis, and enhancing cancer cell proliferation [5]. Hypoxic EVs are reported to impede antitumor immunity, thereby fostering cancer progression [48]. Tumor-derived microvesicles promote the expansion of regulatory T cells and induce apoptosis in tumor-reactive activated CD8^+^ T cells, and is a key promoter of distant metastasis [49, 50]. EVs are well-suited for targeted drug delivery, which may affect ICI responses, and loading them with therapeutic agents can enhance immunotherapy efficacy [51]. Additionally, this study did not assess the effects of IL-2 or its receptor blockade, nor did it examine CD4^+^ T cell depletion using in vivo tumor models. Future studies are required to evaluate the systemic and local effects of HIF-PH inhibitors and IL-2 induction on tumor-associated immune cells, including macrophages and fibroblasts [52, 53].

In conclusion, our study demonstrates that HIF-PH inhibitors can be useful to activate the antitumor immune response, especially in combination with ICIs, for the treatment of MSS CRC.

## Supplementary Information

Below is the link to the electronic supplementary material.

Supplementary file1 (PDF 4528 kb)

Supplementary file2 (DOCX 35 kb)

Supplementary file3 (PDF 223 kb)

## Data Availability

Figure 5F Kaplan–Meier shows progression-free survival of patients with MSS colon cancer classified by stable MSI and the level of HIF-1 expression: high (red) and low (black), measured with Affymetrix arrays (IDs: 220946_s_at). Data were from GSE143985 in GEO database.

## Abbreviations

CKD: Chronic kidney disease
CRC: Colorectal cancer
CSCs: Cancer stem cells
FIH: Factor inhibiting HIF
GZMB: Granzyme B
HIF: Hypoxia-inducible factor
HIF-PH: HIF-prolyl hydroxylase
HPF: High-power field
ICIs: Immune checkpoint inhibitors
IFN-γ: Interferon-gamma
MFI: Mean fluorescence intensity
MMR: Mismatch repair
MSI: Microsatellite instability
MSS: Microsatellite stable
OS: Overall survival
PFS: Progression-free survival
TILs: Tumor-infiltrating lymphocytes
TME: Tumor microenvironment
VHL: Von Hippel-Lindau
WBC: White blood cell
