# Supplementary material for: HIF-PH inhibitors induce pseudohypoxia in T cells and suppress the growth of microsatellite stable colorectal cancer by enhancing antitumor immune responses
Source: Cancer Immunol Immunother. 2025 May 9;74(7):192. doi: 10.1007/s00262-025-04067-3 (PMC12064516; doi:10.1007/s00262-025-04067-3)
Supplement: Supplementary file 1 — Supplementary file1 (PDF 4528 kb) [file 262_2025_4067_MOESM1_ESM.pdf]

# Supplemental Figure 1

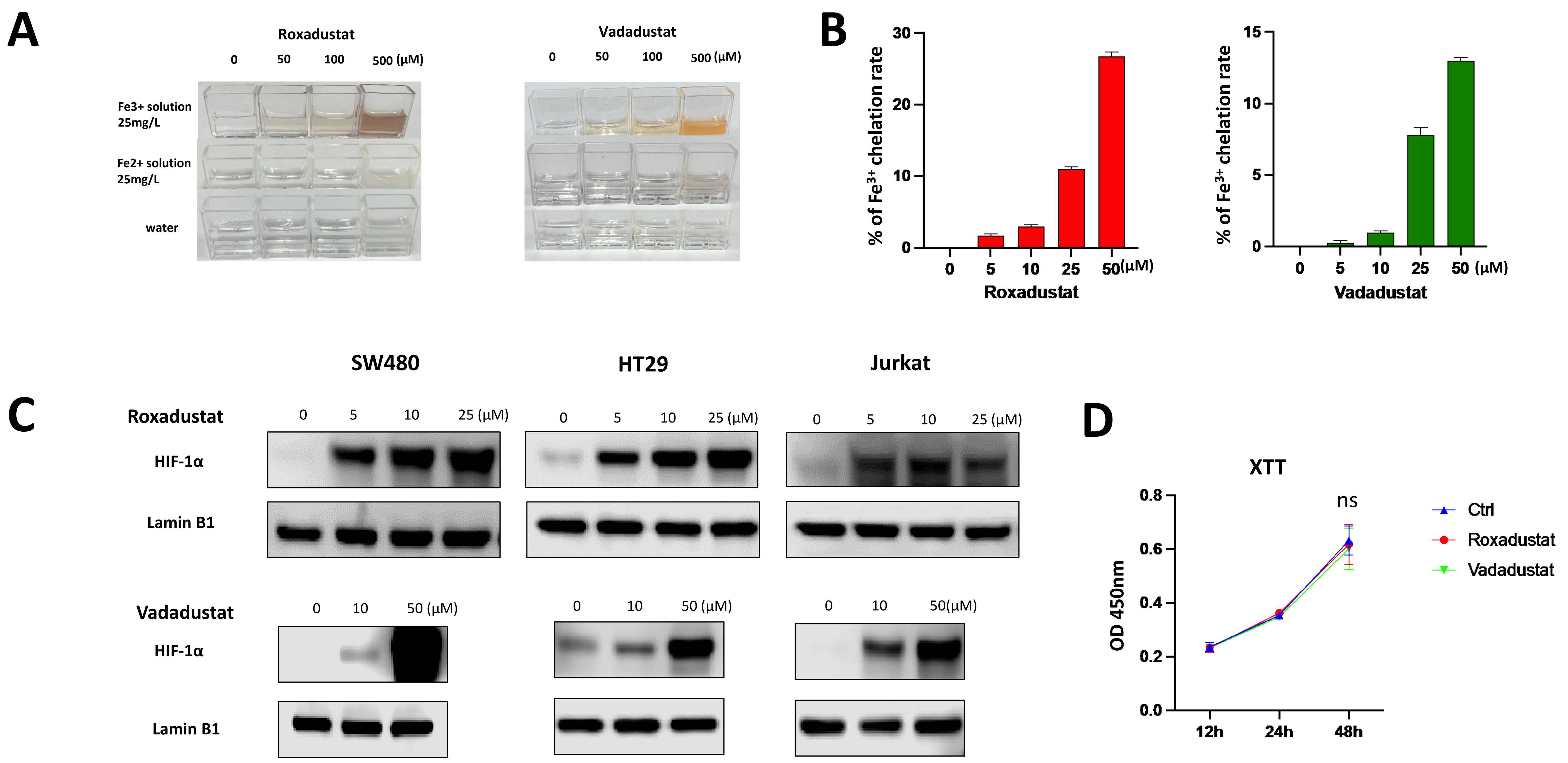


Fig. S1. HIF-PH inhibitors with iron chelating ability increase HIF-1α. (A) The basic chelating abilities of HIF-PH inhibitors were examined by standard $Fe^{2+/3+}$ solution and water. The color of the liquid changed according to iron chelation. (B) The data were obtained by DPM-$Fe^{3+}$ reader through sulfosalicylic acid visual colorimetric method. Roxadustat and Vadadustat were added to the standard $Fe^{3+}$ solution (25 mg/L). (C) SW480 HT29 and Jurkat were treated with different HIF-PH inhibitors for 48h. HIF-1α level was measured using western blotting. (D) XTT assay of Colon26 cells treated with 25 μM Roxadustat and Vadadustat at different time points. The results are presented as a representative experiment performed in triplicate.

# Supplemental Figure 2

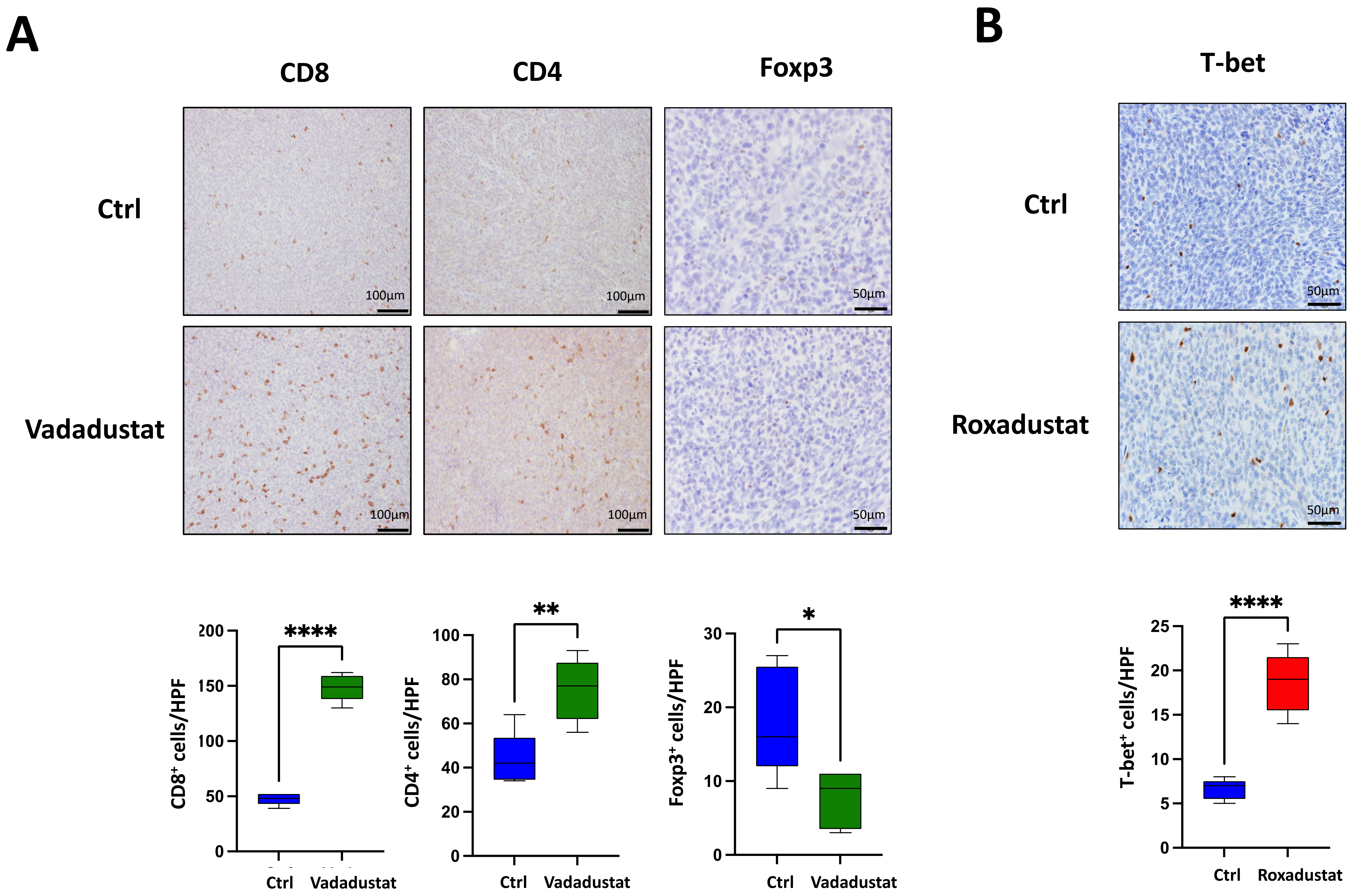


Fig. S2. Vadadustat promotes CD4$^{+}$ and CD8$^{+}$ T cell infiltration and reduce regulatory T cell populations. (A) Representative images and positive cell number of CD8$^{+}$, CD4$^{+}$ and Foxp3$^{+}$ cells stained from tumor tissues of control and Vadadustat group mice (scale bar,100 and 50 μm; quantitative data, blow; n=5). (B) Representative images and positive cell number of T-bet+ cells stained from tumor tissues of control and Roxadustat group mice (scale bar, 50 μm; quantitative data, blow; n=5). Student's t test. *P < 0.05, **P < 0.01, ***P < 0.001.

# Supplemental Figure 3

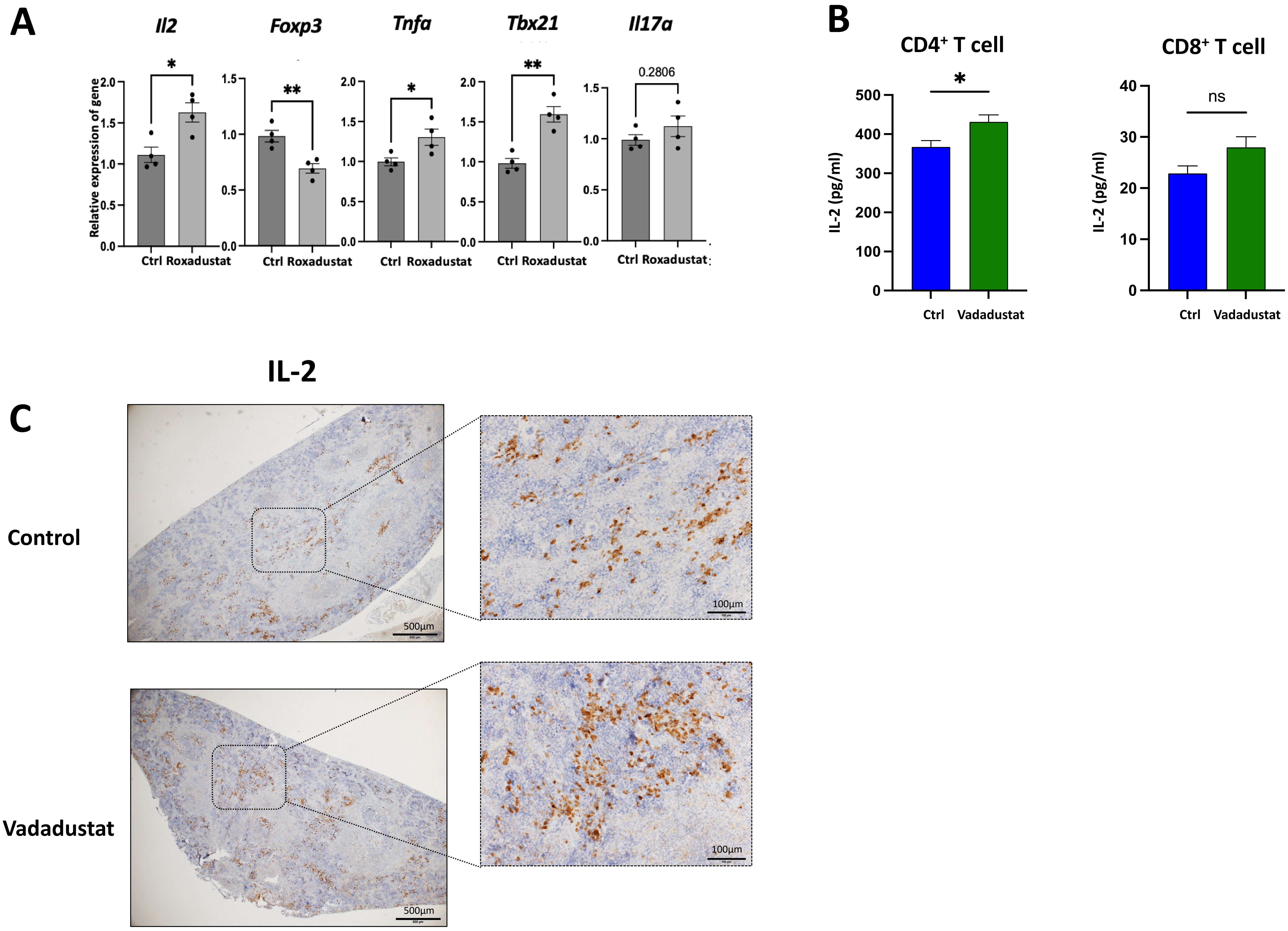


Fig. S3. HIF-PH inhibitors activate CD4⁺ T cell. (A) RT-qPCR results of isolated CD4⁺ T cell were stimulated with 5µg/ml ConA and treated with 5µM Roxadustat or 50ng/ml recombinant IL2 for 12h. (B) Concentrations of IL-2 in the supernatant of indicated murine spleen derived cells stimulated with 5µg/ml ConA and treated with 25µM Vadadustat for 48h. (C) Representative images of IL-2 protein in tumor-bearing mice spleen of control and Vadadustat group mice (scale bar, 500 µm; magnification scale bar, 100 µm). The results are presented as the means ± S.E.M. of a representative experiment performed in triplicate. Student's t test. *P < 0.05, **P < 0.01, ***P < 0.001.

# Supplemental Figure 4

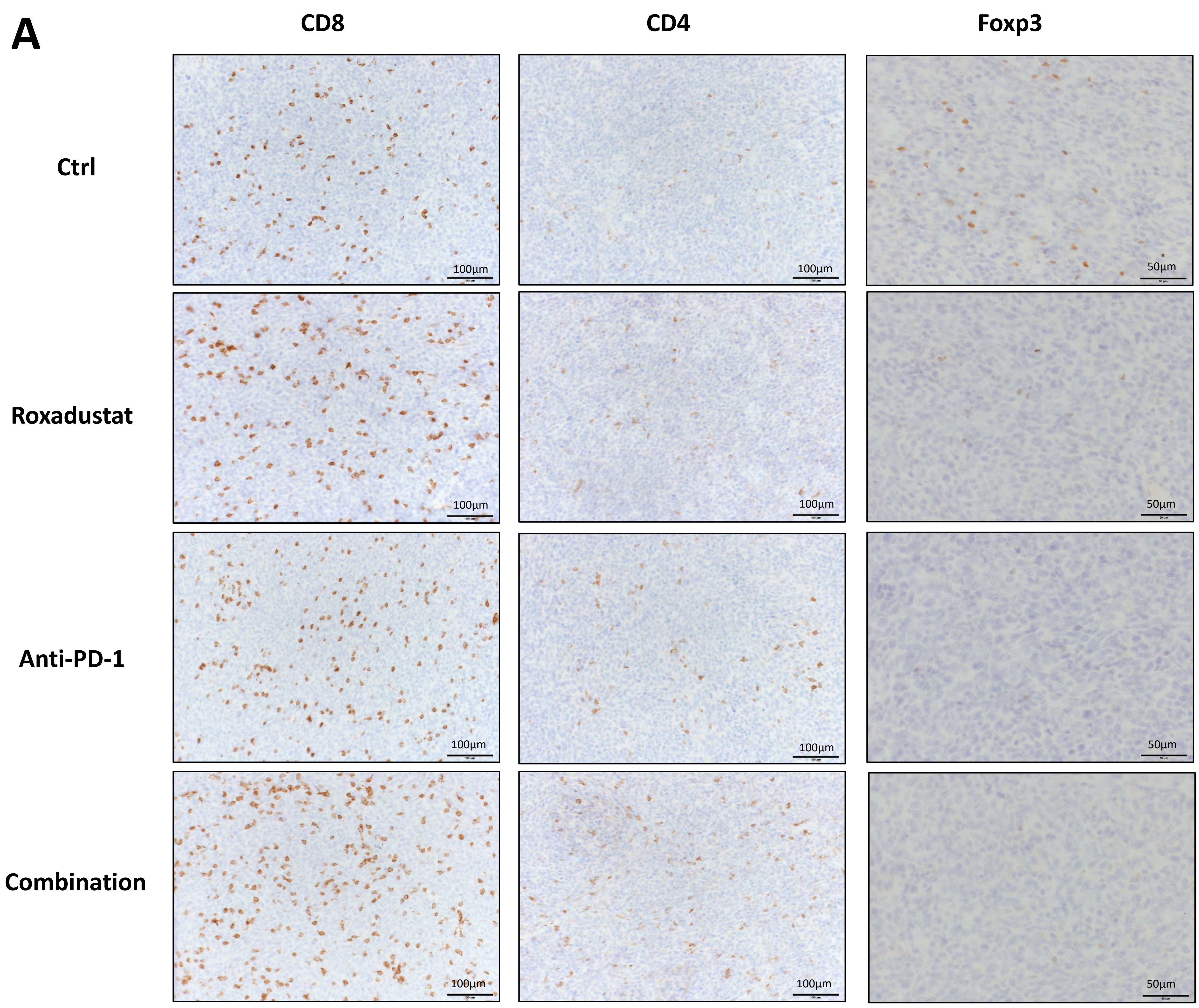


Fig. S4. Roxadustat and anti-PD-1 combination therapy increases TILs. (A) Representative images of indicated markers in the tumors of 4 groups mice (scale bar,100 and 50 μm).

# Supplemental Figure 5

A

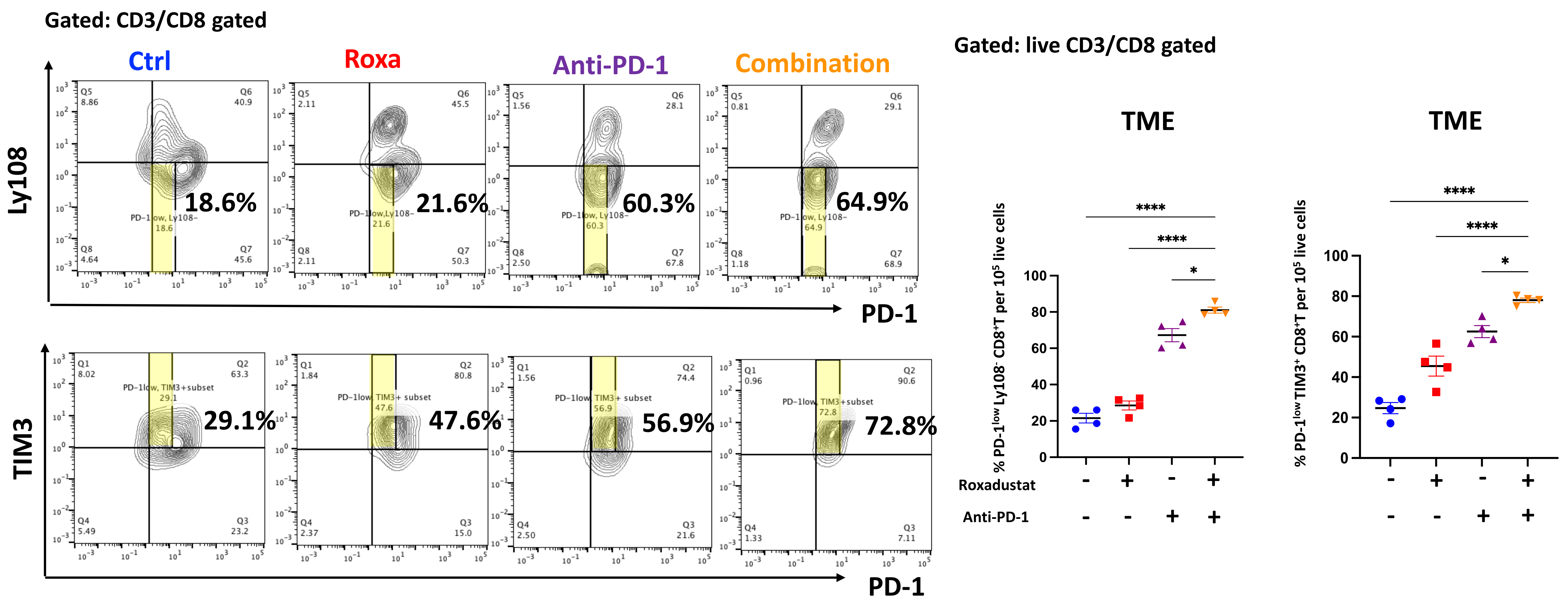


B

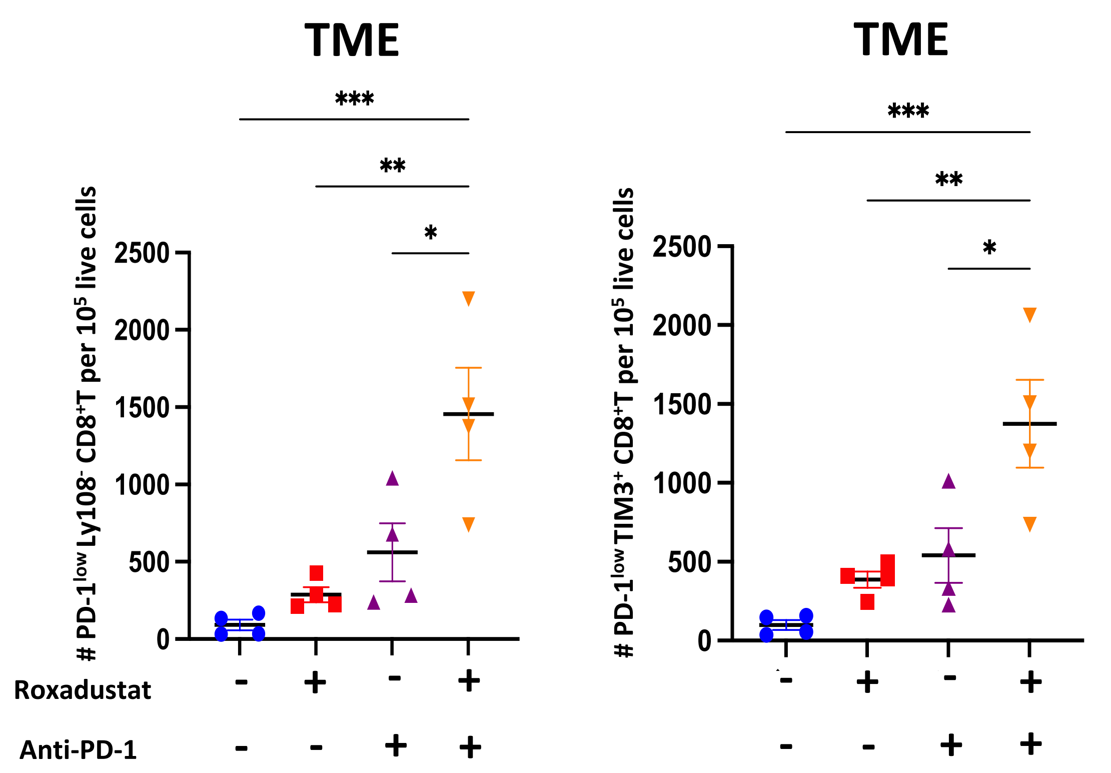


C

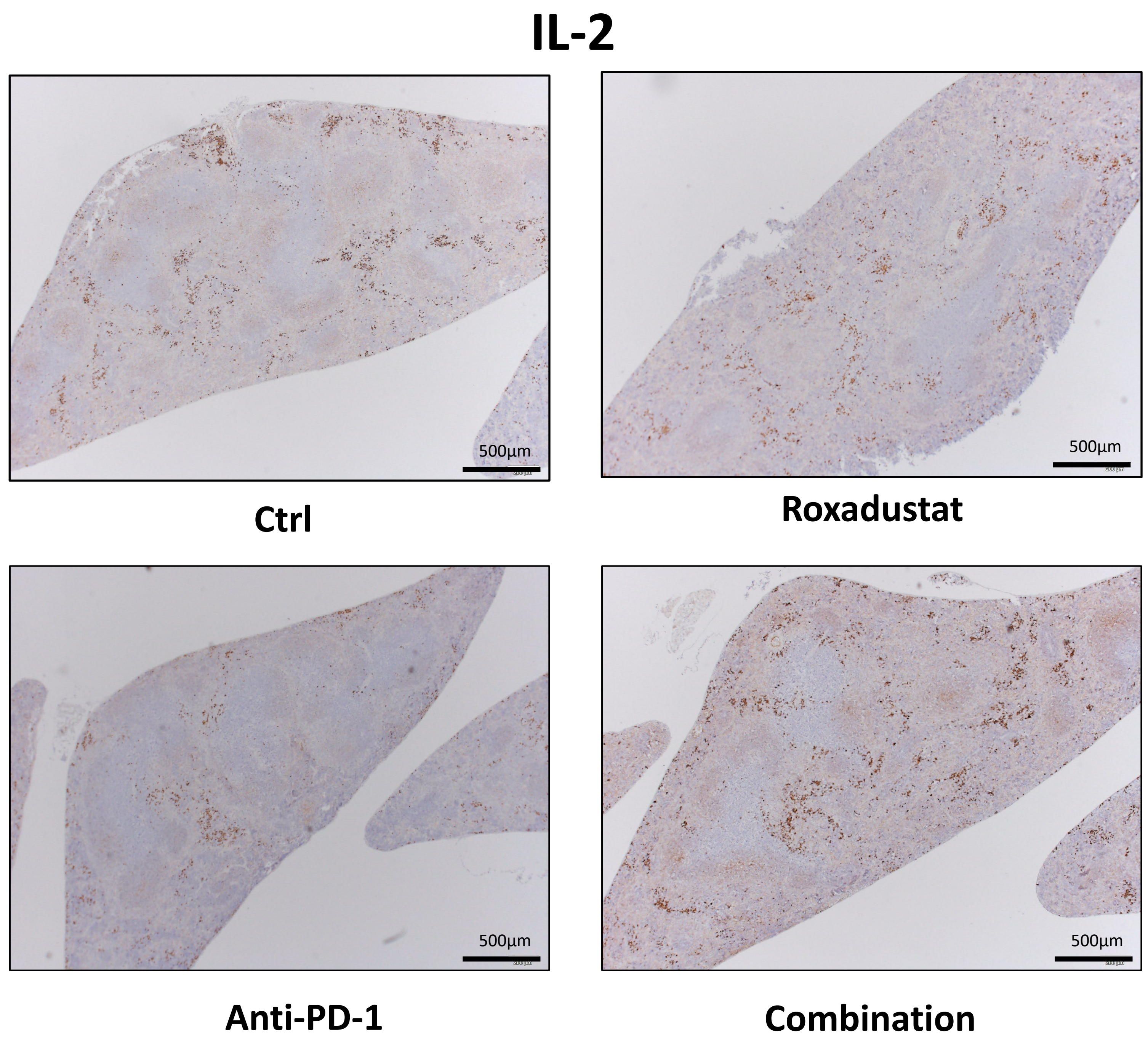


Fig. S5. Synergistic increase of effector-like CD8$^+$T-cell population by Roxadustat combined with anti-PD-1 antibody therapy. (A) TILs from tumor-bearing mice were examined for expression of PD-1$^{low}$, Ly108- and TIM3$^+$ (representative flow plots, left; quantitative data, right; n=4). (B) Number of the indicated CD8$^+$T cells collected from TME of 4 groups. (C) Representative images of IL-2 in the spleens of 4 groups tumor-bearing mice (scale bar,500μm). The results are presented as the means ± S.E.M. of a representative experiment performed in triplicate. ANOVA followed by Tukey's multiple comparison test was applied. *P < 0.05, **P < 0.01, ***P < 0.001.

# Supplemental Figure 6

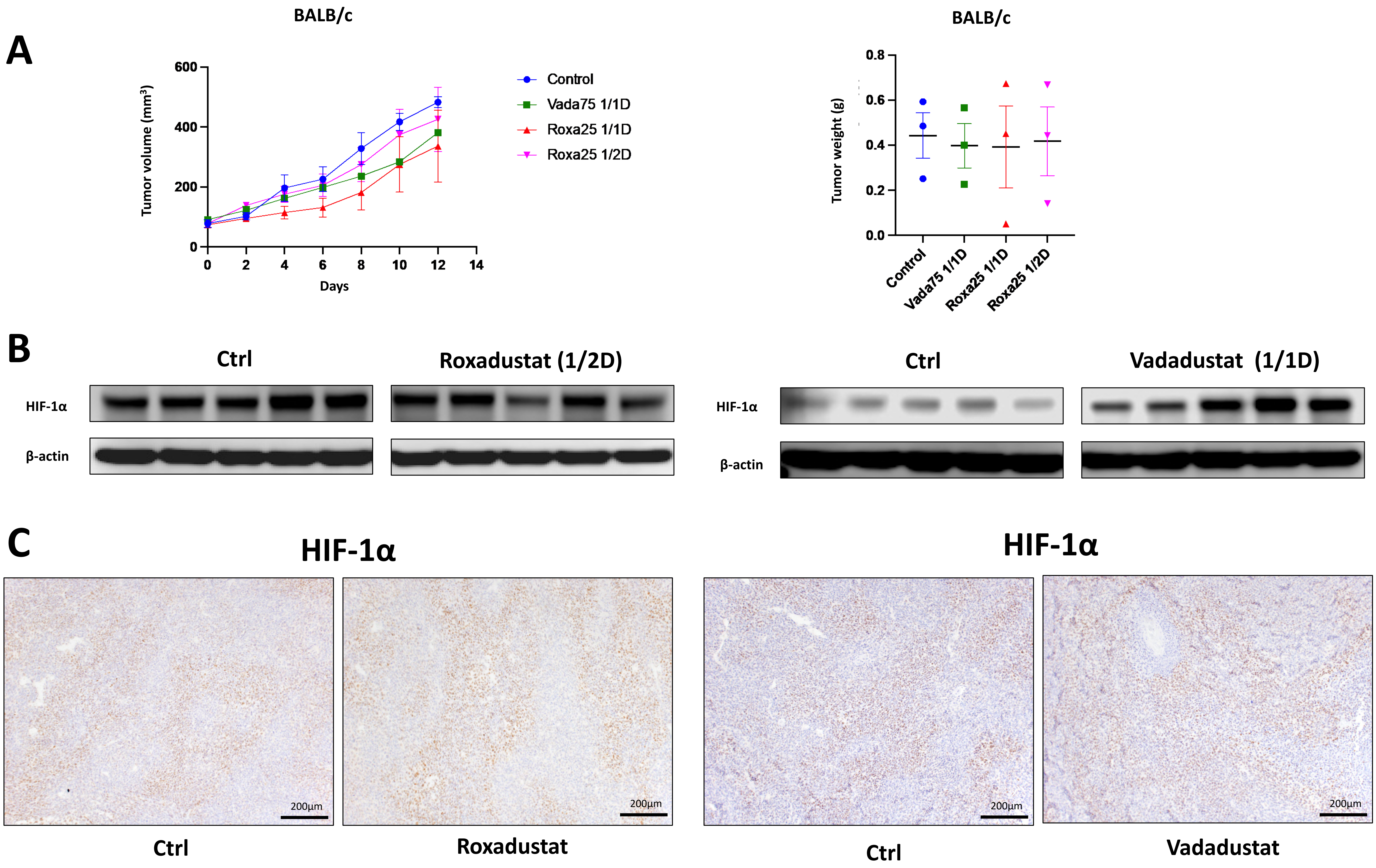


Fig. S6. (A) 7 days post Colon26 injection, BALB/c were randomized into four groups: (i) Control; (ii) Roxadustat: 25 mg/kg. every other day; (iii)Vadadustat: 75 mg/kg. every day; (iv)Roxadustat: 25 mg/kg. every day. PBS and Roxadustat/Vadadustat were administered via oral. Tumor volumes and weights of BALB/ were measured at the indicated day(n = 3). (B-C) 7 days post Colon26 injection, BALB/c were randomized into two groups: (i) Control; (ii) Roxadustat/Vadadustat; PBS and Roxadustat/Vadadustat were administered via oral route (Roxadustat: 50 mg/kg. every other day. Vadadustat: 150 mg/kg. every day). HIF-1α expression of Western Blot and IHC were measured. (n = 5). Values are presented as the mean ± SEM.

# Supplemental Figure 7

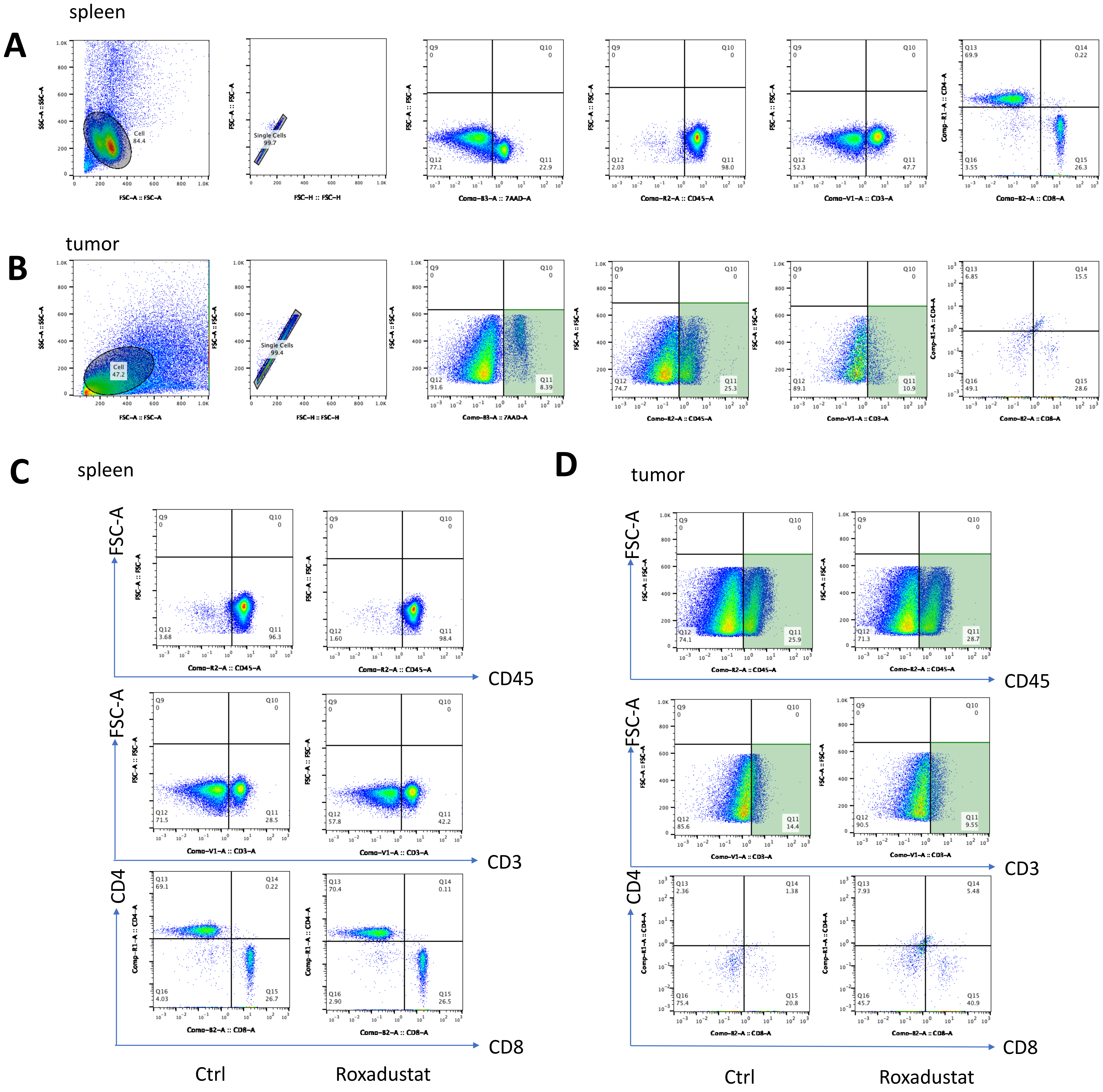


Figure S7:Representative gating strategies and panels of flow cytometry in spleen(A,C) and tumor(B,D).
