# Supplementary material for: HIF-PH inhibitors induce pseudohypoxia in T cells and suppress the growth of microsatellite stable colorectal cancer by enhancing antitumor immune responses
Source: Cancer Immunol Immunother. 2025 May 9;74(7):192. doi: 10.1007/s00262-025-04067-3 (PMC12064516; doi:10.1007/s00262-025-04067-3)
Supplement: Supplementary file 2 — Supplementary file2 (DOCX 35 kb) [file 262_2025_4067_MOESM2_ESM.docx]

**Supplemental Information**

**Materials and Methods**

**Regents**

Roxadustat (FG-4592) was purchased by Astellas Pharma Inc (Tokyo, Japan) and Selleck chemicals (Houston, TX, USA). Vadadustat (AKB-6548) was purchased by Mitsubishi Tanabe Pharma Corporation (Osaka, Japan) and Selleck chemicals (Houston, TX, USA). Antibodies used in this study are listed in Supplemental Table S1.

**Cell lines**

The murine cell line of colon cancer (colon26) and human Jurkat cell were purchased from the Japanese Collection of Research Bioresources (JCRB) Cell Bank (Osaka, Japan). The human colon adenocarcinoma HT29 and SW480 cells were purchased from American Type Culture Collection (Manassas, VA, USA). Colon26 and Jurkat cells were cultured in RPMI 1640 complete medium (Sigma-Aldrich, USA). HT29 and SW480 were cultured in DMEM (high) complete medium (Sigma-Aldrich, USA). Complete mediums were supplemented 10% fetal bovine serum (FBS) (HyClone, USA), 100 μM glutamine, 100 U/ml penicillin, 100 μg/ml streptomycin.

**Cell isolation and culture**

Splenocytes were harvested from fresh spleens after dispersing into cell-suspensions and blood lysis buffer. CD4^+^ and CD8^+^ T cells were isolated from splenocyte-suspensions (purity and %alive cells > 95%) using CD4^+^ and CD8a^+^ T cell isolation kit (Miltenyi Biotec, Bergisch Gladbach, Germany) according to instructions supplied by the manufacturer, respectively. Cells (2× 10^6^cells/mL) were cultured in RPMI 1640 complete medium. Recombinant mouse IL2 (BioLegend) and Ultra-LEAF purified anti-mouse IL2 antibody (BioLegend) were used for IL2 supplement and blockade.

**Cell proliferation assay**

The XTT assay (Cell proliferation kit II, Roche Mannheim, Germany) was used to assess cell proliferation. The cells were incubated with or without HIF-PH inhibitors for 48 hours at 37°C in mediums, supplemented 3% fetal bovine serum (FBS) (HyClone, USA), 100 μM glutamine, 100 U/ml penicillin, 100 μg/ml streptomycin. To maintain T cell viability, 1 μg/mL anti-CD3 and 3 μg/mL anti-CD28 antibodies (ThermoFisher, USA) were added when culturing murine derived CD8^+^ T cell. The XTT reagent mixture was added to each well according to the manufacturer’s instructions for 2 h of incubation at 37°C and 5% CO2 in a humidified incubator, and then each well was measured using a microplate reader between 450 nm and 690 nm. We repeated each experiment at least 3 times and seeded the cells in 96-well plates as follows; murine derived CD8^+^ T cell and Jurkat (2.0 × 10^4^ /well), SW480, HT29 and colon26 (5.0 × 10^3^ /well).

**Mice tumor model**

Male BALB/c and BALB/c-nude mice were purchased from Japan SLC, Inc. (Hamamatsu, Japan) and housed and bred in temperature-controlled and sterile environment of Department of Animal Resources, Okayama University (Okayama, Japan). The experiments began when the mice were 6-8 weeks old. Cultured colon26 tumor cells were harvested and re-suspended in phosphate-buffered saline. Viable colon26 cells (5×10^6^) were injected subcutaneously into the dorsal skin of mice. After 7 days, mice were randomly divided into two groups (n = 5 and 6). Roxadustat (50 mg/kg every other day) or Vadadustat (150 mg/kg every day) were orally administered by using a probe. The dosage was determined based on previous studies [39-43]. Tumor size and body weight were also measured for two weeks. The control group received PBS orally administered orally in the same manner. At the end of the experiment, the animals were sacrificed, and the tumors were collected for weight measurement, histological analysis or flow cytometry. Tumor length and width were measured using a caliper, and tumor volume was calculated using the following formula: Volume = (width)^2^ × length/2

All experiments involving animals were conducted according to the ethical policies and procedures approved by The Animal Care and Use Committee of Okayama University (Approved No. OKU-2021900, OKU- 2022723, OKU- 2022879, OKU- 2023535). The experimental endpoint was determined based on tumor size measurements, with health monitoring conducted three times per week over a four-week period. If the tumor diameter exceeded 20 mm (corresponding to a volume below 4000 mm³) or if the animals exhibited significant weight loss (≥20% of their initial body weight), reduced mobility, or other signs of deteriorating health, the experiment was immediately terminated, and humane euthanasia was performed. Animals were housed under specific pathogen-free conditions, maintained on a 12-hour light/dark cycle, and provided ad libitum access to food and water throughout the study.

**Immunohistochemistry**

Tumor specimens were deparaffinized with xylene and gradually rehydrated with ethanol. Endogenous peroxidase activity was blocked by 3% hydrogen peroxide at room temperature for 10 min. For antigen retrieval, sections were submerged in 10 mM citrate buffer (pH 6.0) or 5 mM EDTA solution (pH 8.0) and microwaved (700 W) continuously for 25 min in a pressure cooker. After cooling，protein blocking buffer(Dako, Santa Clara, CA) were used and then samples were incubated with primary antibodies at 4 ℃ overnight. The indirect avidin-biotin-peroxidase method was applied, using the appropriate secondary antibodies, for 30 min at room temperature and visualized with DAB (Dako, Santa Clara, CA) according to the manufacturer’s instructions. Finally, sections were counterstained with hematoxylin, dehydrated, and mounted. Images were acquired using an Olympus BX43 light microscope connected to a DP73 digital camera (Olympus). Three pictures of regions were selected randomly from each sample to avoid selection bias. The stained cells were counted within these regions, and the total number of CD8^+^, CD4^+^, and Treg positive cells was manually counted. Antibodies for immunohistochemistry used in this study were shown in Supplemental Table S1.

**Flow cytometry**

To analyze tumor-infiltrating immune cells, tumor tissues were resected from mice individually, 21 days after tumor inoculation (14 days after HIF-PH inhibitor treatment). The tumors were minced, meshed by gentle MACS Dissociator (Miltenyi Biotec) and removed blood cell by lysis buffer. Cells were passed through nylon mesh and suspended in flow buffer for staining. After Fc blocking with anti-CD16/CD32 antibody, cells were incubated with antibodies of interest or isotype control IgG and cell populations were examined using MACSQunat 2.6 (Miltenyi Biotec, Bergisch Gladbach, Germany). Lymphocytes were gated using Electronic Volume (EV) versus Side Scatter (SS) in a dual parameter gate, and then analyzed the percentage of each cell population among gated lymphocytes. For intracellular staining, cells were fixed in fixation buffer and intracellular antibody was diluted in permeabilization buffers (BioLegend). The number of each leukocyte population per group was determined from the respective percentage and total number of leukocytes. Similarly, spleen cells of tumor-bearing mice were analyzed. Antibodies and gating strategies for flow cytometry used in this study were shown in Supplemental Table S1 and Fig. S7.

**Reverse transcription** **quantitative PCR (RT-qPCR)**

The tissues were homogenized in Trizol Reagent (Thermo Fisher), and total RNA was isolated by using High Pure RNA Isolation Kit (Roche Applied Science, Mannheim, Germany). First-strand cDNAs were synthesized from 2 mg total RNA using High-capacity cDNA reverse transcription kit (Thermo Fisher). RT-qPCR was run on a StepOnePlus system (Thermo Fisher). Gene expression level of interest was normalized using that of GAPDH expression. Taq-man gene expression assays (Thermo Fisher) used in this study were shown in Supplemental Table S2.

**Western blotting**

After 48h incubation, cells were centrifuged. For nuclear protein isolation, NE-PER Nuclear and Cytoplasmic Extraction Kit (ThermoFisher) was used according to manufacturer’ instructions. BCA Protein Assay Kit was used to measure protein concentrations (TaKaRa, Kusatsu, Japan). Equal amounts of protein samples were denatured at 100°C for 10 min with 4×NuPAGE LDS sample buffer (Invitrogen, Waltham, Massachusetts) and 10×Sample Reducing agent (Invitrogen, Waltham, Massachusetts). 15–30 μg of cell lysates were separated on 4%–12% NuPAGE Bis-Tris precast gel (Thermo Fisher Scientific) and transferred to nitrocellulose blotting membranes (GE Healthcare Life science, Chicago, Illinois). The membranes were blocked for 1h at room temperature with 5% skim milk in TBS-T. Each primary antibody was diluted according to the manufacturer’s instructions and incubated 4°C overnight. The membrane was washed with TBS-T and then incubated with horseradish peroxidase-conjugated secondary antibodies at room temperature for 1h. After incubation with a secondary antibody, the membranes were washed and the presence of the target protein was quantified using a C-DiGit Blot scanner (LI-COR Biosciences, Lincoln, Nebraska).

**Measurement of cytokines**

T cells were stimulated with 5 μg/mL Con A and treated with HIF-PH inhibitors for 48h. At indicated time points of cell culture, supernatants were collected and tested. IL-2 levels were determined by using a standard sandwich ELISA (BioLegend) according to the manufacturer's instructions. These ELISAs did not recognize other murine cytokines available.

**Database analysis**

Survival analysis was conducted using the Kaplan-Meier Plotter (https://kmplot.com/) to assess progression-free survival in patients with MSS colon cancer based on HIF1 expression levels. RNA-seq data and clinical data for CRC patients were obtained from the Gene Expression Omnibus (GEO) database GSE143985, for our analysis^1^.

**Statistical analysis**

Statistical analysis results were analyzed by the GraphPad Prism 9.0 software (San Diego, CA) and presented as the mean ± standard error of mean (SEM). Each experiment was carried out in technical and biological triplicate. Student’s t test was used to compare two groups, p < 0.05 was considered statistically significant. F-test utilized for variance analysis between two groups. When p < 0.05 in the F-test, the t test with Welch's correction was used to compare two groups. One-way ANOVA and two-way ANOVA were used to determine multiple group comparisons. A value of p<0.05 was deemed statistically significant.
