# Supplementary material for: HIF-PH inhibitors induce pseudohypoxia in T cells and suppress the growth of microsatellite stable colorectal cancer by enhancing antitumor immune responses
Source: Cancer Immunol Immunother. 2025 May 9;74(7):192. doi: 10.1007/s00262-025-04067-3 (PMC12064516; doi:10.1007/s00262-025-04067-3)
Supplement: Supplementary file 3 — Supplementary file3 (PDF 223 kb) [file 262_2025_4067_MOESM3_ESM.pdf]

Supplemental Table 1

Table S1

Antibodies used in this study

	**Western blot**	
**Protein name**	Dilution	Company
**Primary antibody**		
**HIF-1α**	1:1000	CST#36169
**Lamin B1**	1:1000	CST#13435
**Secondary antibody**		
**Goat anti-rabbit IgG HRP**	1:2000	CST #7074
	**Immunohistochemistry**	
**Primary antibody**		
**CD8**	1:250	CST#98941
**CD4**	1:4000	abcam#ab237722
**Foxp3**	1:100	Biolegend#126402
**IL2**	1:100	Biolegend#503802
**Secondary antibody**		
**Goat anti-rabbit IgG HRP**	Undiluted	Nichirei #414341
	**Flowcytometry**	
**Purified CD16/32(93)**	1:100	Biolegend#101302
**APC/Cy7-CD45(30-F11)**	1:100	Biolegend#103116
**FITC-CD45(S18009F)**	1:100	Biolegend#157214
**Pacific Blue-CD3(17A2)**	1:100	Biolegend#100214
**APC/Cy7-CD3(17A2)**	1:100	Biolegend#100222
**APC-CD4(GK1.5)**	1:100	Biolegend#100412
**APC-CD8(53-6.7)**	1:100	Biolegend#100712
**FITC-CD8(53-6.7)**	1:100	Biolegend#100706
**PE-Foxp3(MF-14)**	1:100	Biolegend#126404
**APC-CD25(3C7)**	1:100	Biolegend#101910
**FITC-IFN-γ(XMG1.2)**	1:50	Biolegend#505806
**PE-Granzyme B(QA16A02)**	1:20	Biolegend#372208
**PE/Cy7-IL2(JES6-5H4)**	1:20	Biolegend#503832
**APC-CD69(H1.2F3)**	1:100	Biolegend#104514
**Brilliant Violet-PD1(29F.1A12)**	1:100	Biolegend#135218
**PE-TIM3(RMT3-23)**	1:100	Biolegend#119704
**APC-Ly108(330-AJ)**	1:100	Biolegend#134610
**Zombie Yellow-Fixable Viability Dye**	1:500	Biolegend#423104
**7AAD-Viability dye**	1:100	Biolegend#420404

Supplemental Table 2

Table S2	
**Taqman gene expression assays used for RT-qPCR.**	
**RT-qPCR**	
**Gene**	Taqman gene expression assay kit
**GAPDH**	Mm 99999915_g1
**IFN-γ**	Mm 01168134_m1
**granzyme B**	Mm00442834_m1
**CXCL9**	Mm 00434946_m1
**CXCL10**	Mm 00445235_m1
**IL2**	Mm 00434256_m1
**TNFα**	Mm00443258_m1
**granzyme A**	Mm01304452_m1
**perforin**	Mm00812512_m1
**IL10**	Mm 00439614_m1

Supplemental Table 3

Table S3		Ctrl		Roxadustat		Vadadustat	
WBC	10^2/uL	77.00	±11.51	136.00	±27.33	112.40	±20.03
RBC	10^4/uL	921.17	±134.04	1061.40	±90.75	1022.00	±64.48
HGB	g/dL	14.9	±2.18	17.74	±1.87	17.24	±1.03
HCT	%	41.08	±5.99	48.02	±3.8	47.26	±2.68
MCV	fL	44.58	±0.27	45.28	±0.44	46.26	±0.55
MCH	pg	16.17	±0.23	16.68	±0.48	16.88	±0.32
MCHC	g/dL	36.27	±0.58	36.90	±1.35	36.48	±0.36
PLT	10^4/uL	50.58	±34.51	53.22	±26.05	66.98	±9.17
RDW	%	14.05	±0.27	14.10	±0.47	14.84	±0.56
PCT	%	0.2	±0.13	0.22	±0.10	0.29	±0.04
MPV	fL	4.05	±0.39	4.22	±0.23	4.30	±0.51
PDW	%	15.57	±0.76	15.80	±0.33	15.98	±0.78

Table S3: Blood tests were performed after 14 days of treatment for mice without tumor (n=5). The results are presented as the means ± S.E.M. of a representative experiment performed in triplicate.
